# The prevalence, patterns, and antifungal drug resistance of bloodstream infection-causing fungi in Sichuan Province, China (2019–2023): a retrospective observational study using national monitoring data

**DOI:** 10.3389/fmicb.2025.1616013

**Published:** 2025-07-07

**Authors:** Jin Deng, Ya Liu, Ling Shu, Dan Zhou, Yi Xie, Ying Ma, Mei Kang

**Affiliations:** ^1^Department of Laboratory Medicine, West China Hospital, Sichuan University, Chengdu, China; ^2^Clinical Laboratory Medicine Research Center, West China Hospital, Sichuan University, Chengdu, China; ^3^Sichuan Clinical Research Center for Laboratory Medicine, Chengdu, China

**Keywords:** fungal bloodstream infections, antifungal susceptibility, distribution characteristics, epidemiology, public health

## Abstract

**Background:**

The rising global burden of invasive fungal infections and the growing issue of antifungal resistance present critical public health threats. By using multicenter surveillance data from Sichuan Province, we conducted the largest five-year study on fungemia to date. Our objective was to gain insights into regional differences in the distribution and resistance patterns of fungal pathogens.

**Methods:**

We performed a retrospective analysis of fungal bloodstream infections (BSIs) from 31 hospitals (2019–2023). Integrated clinical and laboratory data were analyzed using WHONET 5.6 to assess resistance patterns, and Microsoft Excel (with PivotTable functionality) was used to analyze epidemiological trends.

**Results:**

Annual fungal isolations increased steadily over the study period. *Candida* species accounted for 88.7% (1,805/2,034) of the bloodstream isolates, with *C. albicans* being the most common (38.4%, 694/1,805). The majority of patients were men (58.6%, 1,191/2,034) and aged 46 years or older (80.0%, 1,627/2,034). Intensive care unit (ICU) cases accounted for 36.8% (748/2,034) of the total. *C. albicans* showed the highest fluconazole susceptibility (91.2%, 633/694). Both *C. albicans* and the *C. parapsilosis* complex maintained >80% voriconazole susceptibility, followed by the voriconazole wild-type *C. glabrata* complex (69.3%). *C. tropicalis* exhibited high resistance to fluconazole (36.2%, 21/58) and voriconazole (34.8%, 20/58). *Cryptococcus* spp. displayed non-wild-type rates to amphotericin B (8.7%), flucytosine (5.8%), fluconazole (8.7%), voriconazole (8.0%), and itraconazole (4.1%). Different hospital types isolated varying fungal species. While *C. albicans* was the predominant species in 83.9% (26/31) of the hospitals, pediatric specialty centers exhibited distinct microbiological profiles, showing the highest isolation rates of the *C. parapsilosis* complex (χ^2^ = 18.34, *p* = 0.002).

**Conclusion:**

Our research conducted across several centers, revealed significant geographic variations in the spread of fungal diseases and antifungal resistance. It is important to understand local epidemiology to guide antifungal therapy and enhance stewardship programs.

## 1 Background

Bloodstream infections (BSIs) are life-threatening systemic invasions predominantly caused by bacterial or fungal pathogens (Dolatabadi et al., 2024; Najafzadeh et al., 2024). Notably, fungemia in critically ill patients carries serious prognostic implications, with 30-day attributable mortality rates remaining between 35 and 50% despite advancements in diagnostic modalities and targeted therapies (Oren and Paul, 2014; Ruhnke et al., 2018; Arias et al., 2017). Yeasts of the *Candida* genus are the predominant etiological agents of invasive fungal diseases (IFDs) in hospitalized populations, accounting for 42.7% of all IFDs (Ruiz-Ruigómez et al., 2018). As the first national surveillance system for IFDs, the China Hospital Invasive Fungal Surveillance Network (CHIF-NET) offers valuable epidemiological evidence. However, our analysis of fungemia data from 2019 to 2023 showed that there are regional differences in the distribution of fungal pathogens and antifungal resistance (Enoch et al., 2017). Understanding the epidemiological patterns and *in vitro* antifungal susceptibility characteristics of the causes of fungal BSIs in this region is crucial for clinical diagnosis and treatment. These evidence-based findings directly inform clinical decision-making algorithms for selecting empirical antifungal therapies and help optimize provincial-level antimicrobial stewardship programs.

## 2 Materials and methods

### 2.1 Strains and identification of isolates

This retrospective cohort study followed the CHIF-NET protocols to collect fungal BSI data from 31 tertiary care hospitals across Sichuan Province (January 2019–December 2023). Strains of the same type and from the same patient were excluded, and the data of strains with antifungal sensitivity results were included. When duplicate strains had antifungal sensitivity results, the one isolated first was retained. Demographic and microbiological data were collected included gender stratification, age distribution, clinical department classification, fungal speciation, and antifungal susceptibility profiles. All isolates were identified to the species level using matrix-assisted laser desorption ionization time-of-flight mass spectrometry (MALDI-TOF MS, Bruker Corporation, Germany), VITEK MS (Bio-Merieux, France).

### 2.2 Antifungal agents and standards

Antifungal susceptibility testing was conducted using validated commercial microdilution systems: Sensititre YeastOne (Thermo Fisher Scientific, United States) and ATB FUNGUS 3 (bioMérieux, France). Antifungal susceptibility was determined following the Clinical and Laboratory Standards Institute (CLSI) guidelines. Susceptibility testing for *C. albicans*, the *C. parapsilosis* complex, *C. tropicalis*, and the *C. glabrata* complex (except voriconazole) against fluconazole, voriconazole, anidulafungin, caspofungin, and micafungin was performed using the CLSI M27M44S standards (Clinical and Laboratory Standards Institute, 2022a). Susceptibility of the *C. glabrata* complex to voriconazole, as well as susceptibility of *C. albicans*, the *C. parapsilosis* complex, *C. tropicalis*, and the *C. glabrata* complex to amphotericin B, itraconazole, and posaconazole, was analyzed using the epidemiological cutoff value (ECV) outlined in CLSI M57S (Clinical and Laboratory Standards Institute, 2022b). Similarly, susceptibility of *Cryptococcus* spp. to amphotericin B, flucytosine, fluconazole, voriconazole, and itraconazole was evaluated using the ECV outlined in CLSI M57S. *C. albicans* ATCC 90028 and *C. parapsilosis* ATCC 22019 were used as antifungal sensitivity quality controls.

### 2.3 Software tools and analytical methods

The data were processed using WHONET 5.6 and Microsoft Excel with PivotTable functionality. In addition, we used SPSS 25.0 software to analyze the data, and the count data were expressed as species (n). The χ^2^ test was performed, and a *p*-value of < 0.05 was considered statistically significant.

## 3 Results

### 3.1 Patient information and departmental distribution

**Figure 1:**
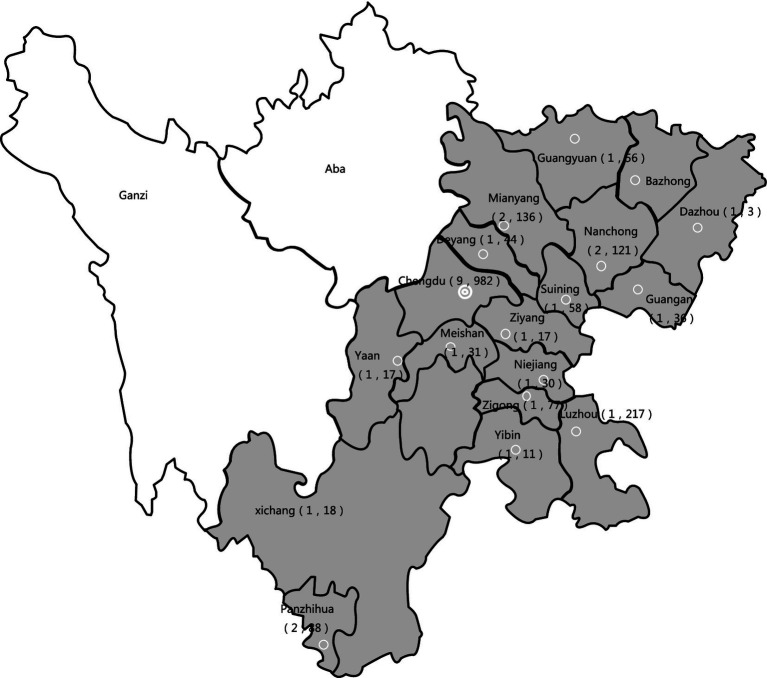
The study’s geographic coverage (18 cities, dark gray). The number of hospitals that participated in the study is indicated by the first number in parentheses under the city name, and the number of isolates gathered is indicated by the second number.

The geographic distribution of the 31 hospitals from 1 January 2019 to 31 December 2023 is shown in Figure 1. Of the 2,034 patients, 58.6% (1,191/2,034) were male individuals and 41.4% (843/2,034) were female individuals. The incidence rates by age group were as follows: 2.7% (55/2,034) for patients aged 0–6 years; 1.3% (26/2,034) for patients aged 7–12 years; 1.1% (22/2,034) for patients aged 13–17 years; 14.9% (304/2,034) for patients aged 18–45 years; 42.0% (854/2,034) for patients aged 46–69 years; and 38.0% (773/2,034) for patients over 69 years. The majority of the patients were admitted to the ICU (36.8%, 748/2,034), followed by the Department of Internal Medicine (27.7%, 564/2,034), Surgical Departments (15.3%, 312/2,034), Emergency Medicine (8.7%, 177/2,034), Outpatient Clinics (4.0%, 81/2,034), Pediatrics (3.9%, 80/2,034), and other departments (e.g., Gynecology, Reproductive Medicine; 3.5%, 72/2,034).

### 3.2 Strain isolation

Longitudinal analysis revealed a significant upward trend in fungal isolations from 2019 to 2023, with an anomalous decline in 2022 potentially associated with COVID-19 containment measures. Among the 2,034 fungemia cases, *Candida* species were the predominant etiological agents (88.7%, *n* = 1,805). *C. albicans* comprised 38.4% (694/1,805), followed by the *C. parapsilosis* complex, *C. glabrata* complex, and *C. tropicalis*. *Cryptococcus* spp. ranked fifth among all isolates, accounting for 8.3% (169/2,034). Detailed species distribution and annual isolate counts are provided in Table 1.

**Table 1:** Distribution of bloodstream infection-causing fungi over a 5-year period.

Species	Overall		Year				
	n	%	2019	2020	2021	2022	2023
***Candida* spp.**	**1805**	**88.7%**					
*C. albicans*	694	38.4%	133	112	186	85	178
*C. parapsilosis* complex	356	19.7%	56	59	106	41	94
*C. glabrata* complex	335	18.6%	49	57	74	41	114
*C. tropicalis*	290	16.1%	46	45	70	22	107
*P. kudriavzevii*	30	1.7%	3	8	6	5	8
*C. lusitaniae*	16	0.9%	/	2	7	/	7
*C. guilliermondii*	15	0.8%	8	4	1	1	1
*W. anomalus*	8	0.5%	2	1	4	/	1
*C. intermedia*	4	0.2%	/	2	1	/	1
*D. rugosa*	2	0.1%	2	/	/	/	/
*D. catenulata*	3	0.2%	/	/	2	/	1
*P. norvegensis*	2	0.1%	/	1	1	/	/
*C. haemulonii*	2	0.1%	1	/	/	/	1
*K. marxianus*	2	0.1%	1	/	1	/	/
*C. inconspicua*	1	0.1%	1	/	/	/	/
*Y. lipolytica*	1	0.1%	/	/	1	/	/
*C. auris*	2	0.1%	/	/	/	/	2
Other	42	2.3%	11	8	11	4	8
***Cryptococcus* spp.**	**169**	**8.3%**					
*C. neoformans*	168	99.4%	29	31	41	20	47
*P. laurentii*	1	0.6%					1
***Talaromyces* spp.**	**49**	**2.4%**					
*T. marneffei*	49	100.0%	11	9	7	8	14
***Trichosporon* spp.**	**11**	**0.6%**					
*T. asahii*	7	63.6%	3	1	1	/	2
*T. ovoides*	2	18.2%	/	1	1	/	/
*T. beigelii*	1	9.1%	/	/	1	/	/
*T. faecale*	1	9.1%	/	/	1	/	/
Total	2034	100.0%	356	341	523	227	587

The bold values in table represent the number of species of each spp.

### 3.3 Antifungal susceptibility testing *in vitro*

The number of tests included in the analysis did not always match the number of strains isolated due to variations in the drug sensitivity kits used at each hospital, which led to the exclusion of certain drugs from the analysis. *C. albicans* showed the highest susceptibility to fluconazole (91.2%), followed by the *C. parapsilosis* complex. *C. albicans* and the *C. parapsilosis* complex exhibited over 80% susceptibility to voriconazole, followed by the voriconazole wild-type *C. glabrata* complex (69.3%). A high percentage of wild-type isolates to itraconazole was observed in the *C. parapsilosis* and *C. glabrata* complexes, as well as in *C. albicans* and the *C. parapsilosis* complex for posaconazole. The resistance rates of *C. tropicalis* to fluconazole and voriconazole were 36.2 and 34.8%, as shown in Table 2. *C. albicans*, the *C. parapsilosis* complex, the *C. glabrata* complex, and *C. tropicalis* showed high susceptibility to amphotericin B, caspofungin, micafungin, and anidulafungin, as shown in Table 3. A total of 169 *Cryptococcus* spp. strains were isolated from 2,034 patients with fungal BSIs. These strains exhibited varying rates of not-wild-type susceptibility to amphotericin B (8.7%), flucytosine (5.8%), fluconazole (8.7%), voriconazole (8.0%), and itraconazole (4.1%), as shown in Table 4.

**Table 2:** *In vitro* susceptibility of the selected pathogenic fungi to azoles.

Species	Fluconazole				Voriconazole				Itraconazole			Posaconazole		
	n	SDD%	S%	R%	n	I%	S/WT%	R/NWT%	n	WT%	NWT%	n	WT%	NWT%
*C. albicans*	693	1.7	91.2	7.1	693	5.5	88.9	5.6	/	/	/	113	93.8	6.2
*C. parapsilosis* complex	356	9.3	76.4	14.3	356	7.3	87.4	5.3	356	96.4	3.6	82	100.0	0.0
*C. glabrata* complex	335	94.3	/	5.7	335	/	69.3	30.7	335	93.4	6.6	49	67.4	32.6
*C. tropicalis*	290	2.8	61.0	36.2	290	10.3	54.9	34.8	290	65.5	34.5	46	32.6	67.4

SDD, susceptible-dose dependent; S, susceptible; I, intermediate; R, resistant; WT, wild-type; NWT, non-wild-type. Data are expressed as numbers and percentages (%) of each species.

**Table 3:** *In vitro* susceptibility of the selected pathogenic fungi to amphotericin B and echinocandins.

Species	Amphotericin B			Caspofungin				Micafungin				Anidulafungin			
	n	WT%	NWT%	n	I%	S%	R%	n	I%	S%	R%	n	I%	S%	R%
*C. albicans*	693	97.8	2.2	369	0.0	96.6	3.4	369	0.0	96.3	3.7	369	0.0	94.6	5.4
*C. parapsilosis* complex	356	99.1	0.9	208	0.0	100.0	0.0	208	0.0	97.9	2.1	208	0.0	100.0	0.0
*C. glabrata* complex	335	98.7	1.3	160	10.9	86.9	2.2	160	3.1	90.6	6.3	160	0.0	91.7	8.3
*C. tropicalis*	290	97.9	2.1	144	0.0	100.0	0.0	144	0.0	100.0	0.0	144	6.9	93.1	0.0

S, susceptible; I, intermediate; R, resistant; WT, wild-type; NWT, non-wild-type. Data are expressed as numbers and percentages (%) of each species.

**Table 4:** *In vitro* susceptibility of *Cryptococcus* spp. to the selected antifungal agents.

Species	Amphotericin B		Flucytosine		Fluconazole		Itraconazole		Voriconazole	
	WT%	NWT%	WT%	NWT%	WT%	NWT%	WT%	NWT%	WT%	NWT%
*Cryptococcus* spp.	91.7	8.3	94.2	5.8	91.3	8.7	92.0	8.0	95.9	4.1

WT, wild-type; NWT, non-wild-type. Data are expressed as percentages (%) of each species.

### 3.4 Changes in resistance to fluconazole and voriconazole

**Figure 2:**
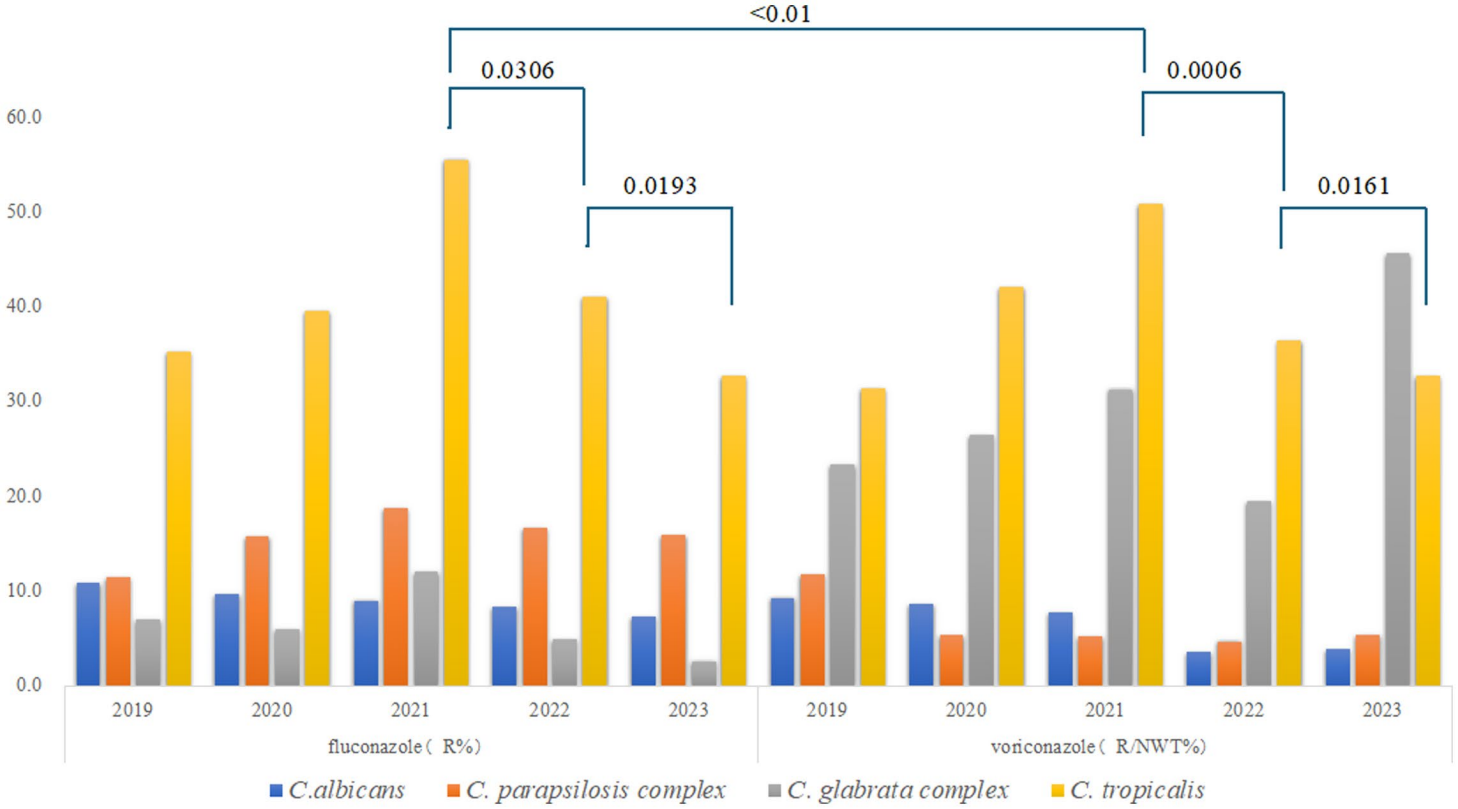
Trends in the resistance of the common *Candida* species to fluconazole and voriconazole. R for Resistant; NWT for Non-wild-type. Different colors represent distinct *Candida* species: blue for *C. albicans*, orange for the *C. parapsilosis* complex, gray for the *C. glabrata* complex, and yellow for *C. tropicalis*.

Among the four common *Candida* species, *C. albicans* exhibited the lowest resistance rates to fluconazole and voriconazole, with both rates declining annually. In contrast, fluconazole resistance in the *C. parapsilosis* complex initially increased but subsequently declined, while voriconazole resistance decreased consistently each year. *C. tropicalis* displayed the highest resistance rates to both antifungal agents in 2021, exceeding 50% (fluconazole: 55.5%; voriconazole: 50.8%; *p* < 0.01), but these rates declined over the following 2 years (*p* < 0.05), as shown in Figure 2.

### 3.5 Differences in the distribution of fungi across hospitals

**Figure 3:**
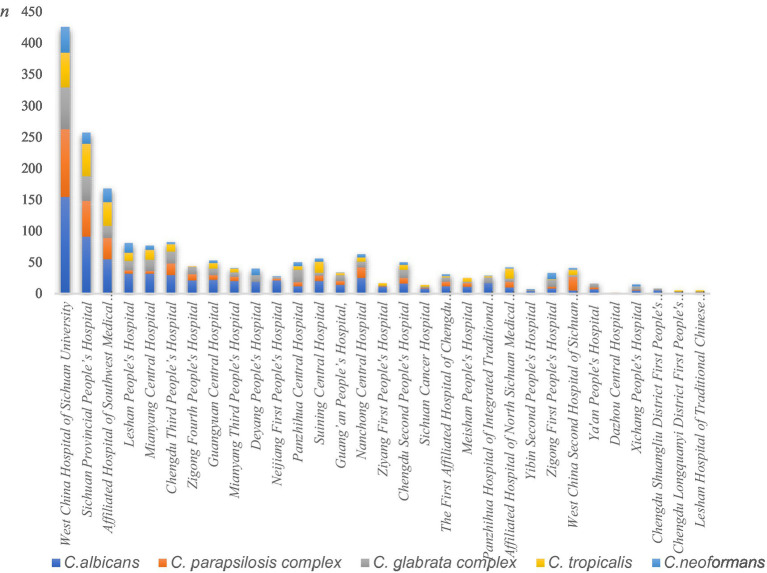
Differences in the distribution of fungal isolates across the 31 hospitals. The Y-axis represents isolate counts, explicitly labeled as “n” (number of isolates). Different colors represent distinct *Candida* species: blue for *C. albicans*, orange for the *C. parapsilosis* complex, gray for the *C. glabrata* complex, yellow for *C. tropicalis*, and light blue for *C. neoformans.*

*C. albicans*, the *C. parapsilosis* complex, the *C. glabrata* complex, and *C. tropicalis* were the predominant species causing fungal BSIs across the 31 hospitals. There was some variation in the ranking of the fungal species isolated across hospitals, although *C. albicans* predominated in most institutions. However, in certain hospitals, such as Panzhihua Central Hospital, Zigong First People’s Hospital, and Xichang People’s Hospital, the *C. glabrata* complex accounted for the highest number of isolates. The highest number of *C. tropicalis* isolates was reported at the Affiliated Hospital of Sichuan North Medical College, while the *C. parapsilosis* complex was the most frequently isolated fungal species at the Second West China Hospital of Sichuan University. Figure 3 shows the differences in the distribution of fungal isolates responsible for bloodstream infection across hospitals.

## 4 Discussion

The number of patients with invasive mycoses is rising due to increasing risk factors such as immunocompromised states, neoplastic diseases, leukemia, and invasive operations (Pappas et al., 2018; Puig-Asensio et al., 2014). The annual number of fungal BSIs reported from 31 hospitals in the Sichuan provincial center of the CHIF-NET increased steadily from 2019 to 2023, with the exception of 2022 when a decline occurred likely due to COVID-19-related disruptions. This is the largest recent study of fungemia in Sichuan. Few other studies in China have conducted province-wide surveillance over a 5-year period, particularly in comparing different types of hospitals. In our study, men accounted for 58.6% (1,191/2,034) of the patients, with 80.0% (1,627/2,034) being middle-aged or elderly patients (>46 years old). Previous studies have shown that fungal BSIs are highly prevalent in middle-aged and elderly populations. In addition, 36.8% (748/2,034) of the cases were from ICUs, followed by the Department of Internal Medicine and Surgical Departments. IFDs in ICUs represent a critical clinical challenge, with a global incidence increasing by 6.8% annually. This rise is largely due to the severity of illness in ICU patients and risk factors such as invasive procedures, prolonged bed rest, and extended antifungal use, all of which contribute to an increased likelihood of infection (Vincent et al., 2009).

*Candida* species dominated the fungal distribution (88.7%, 1,805/2,034). The most common species was *C. albicans*, followed by the *C. parapsilosis* complex, the *C. glabrata* complex, and *C. tropicalis*. This distribution differs from reports in other countries and shows regional variation compared to national data from the CHIF-NET (Xiao et al., 2018a). The pathogenicity of fungal BSIs shifts from *C. albicans* to non-albicans *Candida* species, and there is also variation in the virulence of different *Candida* species (Arendrup, 2013). In this study, non-albicans *Candida* species outnumbered *C. albicans* as causes of BSIs. This shift in pathogen distribution may reflect the widespread use of antifungal agents, which can alter the epidemiology of fungal BSIs (Pfaller et al., 2019). Two strains of *C. auris* were detected in 2023, representing only 0.1% (2/2,034) of the cases. However, *C. auris* is an emerging fungus that can cause serious illnesses and is easily spread among patients in healthcare facilities. The emergence and rapid increase of *C. auris* should be considered a threat. *C. auris* was first discovered in China in 2018 (Wang et al., 2018; Chen et al., 2018). As of December 2023, *C. auris* has been reported in 10 provinces across China. A study reported 312 cases of *C. auris* infections across 18 hospitals. The researchers found significant differences in prevalence between years, with the lowest number of cases occurring in 2020–2021. Notably, the number of infections increased dramatically to 182 in 2023, marking a 450% rise compared to 2022 (33 cases) (Bing et al., 2024). *C. auris* causes serious infections including bacteremia, wound infections, and catheter-associated infections (Tsay et al., 2017), and it can also colonize the urinary tract, respiratory system, digestive tract, and central nervous system (Eyre et al., 2018). The fungus persists in hospital environments and colonizes patients’ skin, posing significant infection control risks. Primarily affecting immunocompromised individuals through nosocomial transmission, *C. auris* carries a high mortality rate (up to 60% in BSI cases) (Boutin and Luong, 2024). Its propensity for causing hospital outbreaks jeopardizes the safety of patients and healthcare workers. On the other hand, *C. auris* is naturally prone to carry antifungal resistance-related genes, and more than 90% of clinical isolates of *C. auris* are resistant to azole antifungal agents (Chowdhary et al., 2023). Resistance to other antifungal agents (e.g., amphotericin B or echinocandins) is also frequently observed, complicating treatment strategies for *C. auris* infections and significantly increasing healthcare costs, compared to other fungal infections.

The isolation rates of different fungi varied across hospitals. For example, the *C. parapsilosis* complex was the most frequently isolated fungal pathogen at the Second Hospital of West China, Sichuan University, accounting for 53.6% (22/41) of cases. This may be because the hospital is a specialized children’s hospital (Yılmaz-Ciftdoğan et al., 2021). The *C. parapsilosis* complex comprises *C. parapsilosis*, *C. metapsilosis*, and *C. orthopsilosis*. Although these species were not individually listed in the Results section of this study, it should be noted that they exhibit differences in antimicrobial susceptibility breakpoints and phenotypic profiles. Similar considerations apply to the *C. glabrata* complex. Unlike in adults, the *C. parapsilosis* complex may be the predominant pathogen causing fungal BSIs in children. Among *Candida* species, the *C. parapsilosis* complex ranks second only to *C. albicans* in biofilm-forming capability among clinically relevant species (Mancera et al., 2021). Biofilms formed by this complex are structurally less complex and thinner than those of *C. albicans*, facilitating their attachment to medical devices (Larkin et al., 2018). In children, the gut is not fully developed, allowing the *C. parapsilosis* complex to enter the bloodstream through damaged mucosal barriers. This leads to infections in children. It can also be transmitted to immunocompromised children by healthcare workers through contact.

An important limitation of our study is the variability in antifungal susceptibility testing methods among the participating hospitals, which may affect the comparability of resistance rates for certain agents. Nevertheless, the data presented in this study can still truly reflect the prevalence of fungal BSIs in Sichuan Province. In this study, the best *in vitro* susceptibility of *Candida* to the antifungal agents analyzed was observed with echinocandins, followed by amphotericin B. Among the azoles, fluconazole (except *C. albicans*) and voriconazole showed sensitivities of no more than 90%. This may be due to the extensive clinical use of fluconazole, which has driven natural selection in *Candida* itself, leading to mutations in certain genes that make it less sensitive to fluconazole (Andes et al., 2006). The *C. parapsilosis* complex showed resistance rates of 14.3 and 5.3% to fluconazole and voriconazole, respectively, both of which were higher than those reported in 20 consecutive years of surveillance data from the SENTRY Antimicrobial Surveillance Program (Pfaller et al., 2019). *C. tropicalis* exhibited resistance rates of 36.2 and 34.8% to fluconazole and voriconazole, respectively, with rates exceeding 50% in 2021. These values are notably higher than those reported by the CHIF-NET (fluconazole 21.0% vs. voriconazole 21.4%) (Xiao et al., 2018a). As with the threat posed by the emergence and rapid spread of *C. auris*, the increasing azole resistance rate of *C. tropicalis* also warrants serious attention. The rate of drug resistance in *C. tropicalis* has been increasing annually, as shown in some studies (Fan et al., 2017). The increase in isolation rates of azole-resistant strains was not associated with the amount of azole antifungal used. However, several mechanisms of resistance to azoles in *C. tropicalis* have been identified, including mutations and overexpression of the ERG11 gene (Morio et al., 2017), mutations and overexpression of the UPC2 gene (Choi et al., 2016), and overexpression of the MDR1 and CDR1 genes (Fan et al., 2019). The observed increase in fluconazole resistance poses a direct threat to the effectiveness of first-line therapy for *C. tropicalis* infections. In regions where resistance rates exceed 10%, Infectious Diseases Society of America (IDSA) guidelines recommend avoiding fluconazole for the empirical treatment of BSIs. Our data suggest that local stewardship programs should prioritize the use of echinocandins or voriconazole in high-risk patients (Pappas et al., 2016). In this study, resistance rates to fluconazole and voriconazole were higher than those observed for itraconazole. Further investigation is needed to determine the underlying reasons for this difference. The IDSA guidelines recommend echinocandins as the best treatment for IFDs because resistance to azole antifungal agents has increased. The European Committee on Antimicrobial Susceptibility Testing (EUCAST) has not established a breakpoint for caspofungin because susceptibility results for caspofungin vary significantly across laboratories. The EUCAST also does not recommend using caspofungin MIC values for clinical evaluation. Some laboratories found that certain *C. glabrata* complex isolates were susceptible to anidulafungin but not to caspofungin when tested using methods such as the E-test and Sensititre YeastOne for sensitization. This discrepancy may be related to the variability of the *in vitro* assay for caspofungin (Arendrup and Pfaller, 2012; Eschenauer et al., 2014). Therefore, the EUCAST recommends using anidulafungin and micafungin as markers for caspofungin susceptibility. Until 2018, when the first case of echinocandin-resistant *C. glabrata* infection was reported in China. The main mechanism of its resistance to echinocandins is a mutation in FKS1/FKS2, and the FKS2 E655K mutation (Fks2 HS1) is a recently identified echinocandin resistance site (Xiao et al., 2018b). Therefore, when testing for caspofungin, the result can be directly reported as “Sensitive.” When an “Intermediate” or “Resistant” result is obtained, it is necessary to confirm the result using the following methods: 1. Additional testing for micafungin or anidulafungin; 2. DNA sequencing analysis, including screening for FKS1 point mutations in all *Candida* species and FKS2 point mutations specifically in *C. glabrata*; and 3. sending the isolate to a reference laboratory for further confirmation. In addition, *Candida* spp. should report resistance to all echinocandins (including caspofungin) if they are resistant to anidulafungin or micafungin or carry an FKS point mutation.

The preferred treatment for *Cryptococcus* infections is amphotericin B (Iyer et al., 2021). In our study, 8.3% (14/169) of *Cryptococcus* strains were classified as non-wild-type for amphotericin B, which is inconsistent with the results of a global drug sensitivity study of 3,590 novel *Cryptococcus* strains conducted by Espinel-Ingroff et al. (2012). A total of five *Cryptococcus* strains exhibited non-wild-type susceptibility to amphotericin B (four strains with an MIC = 1 μg/mL; one strain with an MIC = 2 μg/mL). This reduced susceptibility may reflect prolonged amphotericin B exposure in patients with disseminated cryptococcosis, where antifungal pressure selects for resistant subpopulations (Córdoba et al., 2016). In addition, since most laboratories use Sensititre YeastOne, which is not the gold standard (broth microdilution method), this may result in the observation of higher levels of not-wild-type strains.

Our findings reveal a critical geospatial divergence in both fungal pathogen distribution and resistance profiles. Given the high prevalence of azole-resistant *C. tropicalis* and the emergence of *C. auris*, empirical treatment with echinocandins should be considered for high-risk patients, particularly in settings where resistance rates exceed established thresholds. Accurate knowledge of the regional epidemiology of pathogenic fungi and suspected fungal BSIs is essential for the early selection of appropriate empirical antifungal treatment or intervention, even before definitive pathogenic evidence is obtained.

## Data Availability

The original contributions presented in the study are included in the article/supplementary material, further inquiries can be directed to the corresponding author.
